# Deep Supervised Learning Using Local Errors

**DOI:** 10.3389/fnins.2018.00608

**Published:** 2018-08-31

**Authors:** Hesham Mostafa, Vishwajith Ramesh, Gert Cauwenberghs

**Affiliations:** ^1^Institute for Neural Computation, University of California, San Diego, San Diego, CA, United States; ^2^Department of Bioengineering, University of California, San Diego, San Diego, CA, United States

**Keywords:** backpropagation, local errors, hardware accelerators, supervised learning, biological learning

## Abstract

Error backpropagation is a highly effective mechanism for learning high-quality hierarchical features in deep networks. Updating the features or weights in one layer, however, requires waiting for the propagation of error signals from higher layers. Learning using delayed and non-local errors makes it hard to reconcile backpropagation with the learning mechanisms observed in biological neural networks as it requires the neurons to maintain a memory of the input long enough until the higher-layer errors arrive. In this paper, we propose an alternative learning mechanism where errors are generated locally in each layer using fixed, random auxiliary classifiers. Lower layers could thus be trained independently of higher layers and training could either proceed layer by layer, or simultaneously in all layers using local error information. We address biological plausibility concerns such as weight symmetry requirements and show that the proposed learning mechanism based on fixed, broad, and random tuning of each neuron to the classification categories outperforms the biologically-motivated feedback alignment learning technique on the CIFAR10 dataset, approaching the performance of standard backpropagation. Our approach highlights a potential biological mechanism for the supervised, or task-dependent, learning of feature hierarchies. In addition, we show that it is well suited for learning deep networks in custom hardware where it can drastically reduce memory traffic and data communication overheads. Code used to run all learning experiments is available under https://gitlab.com/hesham-mostafa/learning-using-local-erros.git.

## 1. Introduction

Gradient descent training techniques (Bottou, 1991) have been remarkably successful in training a broad range of network architectures. This success is often attributed to the use of deep architectures with many non-linearity stages (Ba and Caruana, 2014) where backpropagation is used to calculate the direction of weight updates in deep layers. In convolutional networks in particular, multiple cascaded convolutional layers allow simple, lower-level, features to be successively composed into more complex features, allowing networks to obtain highly complex and relevant features from the top convolutional layers (Razavian et al., 2014). Deep convolutional neural networks trained using backpropagation are thus achieving record performance in a variety of large-scale machine vision tasks (Krizhevsky et al., 2012; Simonyan and Zisserman, 2014; LeCun et al., 2015; He et al., 2016; Zagoruyko and Komodakis, 2016; Huang et al., 2016). For deep convolutional networks trained in a supervised setting, the training objective is typically the minimization of classification error at the top network layer. This objective is sometimes augmented by auxiliary objectives defined using the outputs of intermediate classifiers in the network (Szegedy et al., 2014; Lee et al., 2015). These auxiliary objectives provide additional sources of error to deeper layers. Training, however, involves error signals that must propagate backwards from the top layer.

Standard backpropagation is biologically unrealistic for several reasons: the need to buffer network states until errors arrive from the top layer; weight symmetry in the forward and backward passes; and the need to precisely interleave the forward and backward passes. Several biologically-motivated learning mechanisms have been proposed to explain how circuits in the brain are able to learn complex, hierarchical representations. One broad class of these proposals is based on contrastive learning in energy-based models (Xie and Seung, 2003; Bengio and Fischer, 2015; Scellier and Bengio, 2017). In these models, the network is trained to minimize the discrepancy between its equilibrium points when running freely and when observables clamp the values of some units in the network. Weight symmetry is required, though: each synaptic connection from one neuron to another assumes a matching synaptic connection of identical strength in the reverse direction. In Lillicrap et al. (2016) and Baldi et al. (2016), weight symmetry is avoided by using an independent set of fixed random weights to backpropagate errors between the network layers. However, like standard backpropagation, the error signals are non-local. Instead of backpropagating errors layer by layer through the random feedback connections, the networks in Nøkland (2016) and Neftci et al. (2017) directly use a fixed random projection of the top layer error as the error signal in deep layers. Although this permits a single global error signal communicated in common to all layers, is still incurs substantial wait times and memory requirements for the weight updates as a forward pass through the entire network has to be completed before the error signal is available, which requires deep layers to hold their states for the duration of the full forward pass.

We propose a learning approach where weights in any given layer are trained based on local errors that are generated solely based on neural state variables in that layer. These errors are generated directly from the training labels using a classifier with fixed random weights and no hidden layers, and whose input is the neural activations in the layer being trained. Instead of minimizing a global objective function, training thus minimizes many local objective functions. As such this approach compromises one of the core tenets of standard backpropagation: the adjustment of all parameters in concert to minimize a unified objective. Nevertheless, training with local errors still allows a deep network to compose the features learned by lower layers into more complex features in higher layers. This is evidenced by the improvement in accuracy of the random local classifiers in deeper layers. Training with local errors thus retains the hierarchical composition of features, one of the key strengths of deep networks. Our learning approach based on local errors still lags behind backpropagation in terms of test-set accuracy, though.

To implement weight updates based on backpropagation in a biologically inspired network, the pre- or post-synaptic neurons need to buffer the past activity of the pre-synaptic neurons and reproduce this past activity in sync with the corresponding errors arriving from top layers in order to update the weights. This is incompatible with biologically motivated synaptic weight update rules that are typically triggered by pre-synaptic events and depend on the relative timing of pre- and post-synaptic spikes and/or state variables in the post-synaptic neuron. Our learning mechanism bypasses biological implausibility arguments against standard backpropagation by generating errors locally in each layer using fixed random projections. Weight updates could thus be carried out while the synaptic currents in post-synaptic neurons (the neurons receiving the local error signal) still retain a memory of recent pre-synaptic activity. Weight symmetry in the forward and backward passes in standard backpropagation learning is another biologically unrealistic aspect. In our case, the weight symmetry requirement arises in the one-step error backpropagation from the output of the local random classifier to the neurons in the layer being trained. Similar to Lillicrap et al. (2016), we experimented with relaxing this symmetry requirement by using a different set of random, fixed weights to map the classifier error to the error at the layer being trained.

We analyze the implications of the proposed learning approach for the design of custom hardware devices for learning the parameters of deep networks. In the proposed learning approach, there is no explicit backward pass as errors are locally generated and can be used to directly update the weights. We show that our approach drastically reduces memory traffic compared to standard backpropagation in the typical situation when the network weights and activations can not all fit into the compute device memory. We achieve this reduction even despite an increased number of parameters in the network due to the addition of the random local classifier weights in each layer. These weights, however, are fixed allowing them to be generated on the fly using pseudo-random number generators (PRNGs). Only the negligibly small random seeds of the PRNGs for each layer need to be stored.

We discuss related work in section 2. We describe the proposed learning mechanism in section 3 and quantitatively assess the hardware-related computational and memory access benefits compared to standard learning with global objective functions in section 4. We present the results of applying the proposed learning method to standard supervised learning benchmarks in section 5 and compare our learning method's performance to that of the feedback alignment technique (Lillicrap et al., 2016). We present our conclusions and further discussion on the biological plausibility of the proposed learning mechanism in section 6.

## 2. Related work

Training of deep convolutional networks is currently dominated by approaches where all weights are simultaneously trained to minimize a global objective. This is typically done in a purely supervised setting where the training objective is the classification loss at the top layer. To ameliorate the problem of exploding/vanishing errors in deep layers (Hochreiter et al., 2001), auxiliary classifiers are sometimes added to provide additional error information to deep layers (Szegedy et al., 2014; Lee et al., 2015). Unlike our training approach, however, training still involves backpropagating errors across the entire network and simultaneous adjustments of all weights.

Several learning mechanisms have been traditionally used to pre-train a deep network layer-by-layer using local error signals in order to learn the probability distribution of the input layer activations, or in order to minimize local reconstruction errors (Hinton et al., 2006; Hinton and Salakhutdinov, 2006; Bengio et al., 2007; Vincent et al., 2008; Erhan et al., 2010). These mechanisms, however, are unsupervised and the networks need to be augmented by a classifier layer, typically added on top of the deepest layer. The network weights are then fine-tuned using standard backpropagation to minimize the error at the classifier layer. Supervised layer-wise training has been pursued in Bengio et al. (2007), with auxiliary classifiers that are co-trained, unlike the random fixed auxiliary classifiers proposed here. The supervised layer-wise training is used only as a pre-training step, and results are reported after full network fine-tuning using backpropagation from the top classifier layer. Some approaches forego the fine-tuning step and keep the network fixed after the unsupervised layer-wise training phase, and only train the top classifier layer or SVM on the features learned (Ranzato et al., 2007; Lee et al., 2009; Kavukcuoglu et al., 2010). Local learning in Ranzato et al. (2007) and Kavukcuoglu et al. (2010) involves an iterative procedure for learning sparse codes which is computationally demanding. The network architectures in Ranzato et al. (2007), Lee et al. (2009), and Kavukcuoglu et al. (2010) fail to yield intermediate classification results from the intermediate layers. Moreover, their applicability to datasets that are more complex than MNIST is unclear since labels are not used to guide the learning of feature. In more complex learning scenarios with an abundance of possible features, these networks could very well learn few label-relevant features, thereby compromising the performance of the top classifier.

Instead of layer-wise pre-training, several recent approaches train the whole network using a hybrid objective that contains supervised and unsupervised error terms (Zhao et al., 2015). In some of these network configurations, the unsupervised error terms are local to each layer (Zhang et al., 2016). The supervised error term, however, requires backpropagating errors through the whole network. This requirement is avoided in the training approach in Ranzato and Szummer (2008) used to learn to extract compact feature vectors from documents: training proceeds layer by layer where the error in each layer is a combination of a reconstruction error and a supervised error coming from a local classifier. The local auxiliary decoder and classifier pathways still require training, however. Other approaches also make use of a combination of supervised (label-dependent) and unsupervised error signals to train Boltzmann machines as discriminative models (Larochelle and Bengio, 2008; Goodfellow et al., 2013). Learning in Goodfellow et al. (2013), however, is more computationally demanding than our approach as as it involves several iterations to approach the mean-field equilibrium point of the network, and errors are still backpropagated through multiple layers. In Larochelle and Bengio (2008), multi-layer networks are not considered and only a single layer RBM is used.

Several approaches use clustering techniques to learn convolutional layer features in an unsupervised manner (Coates and Ng, 2012; Dundar et al., 2015). A biologically-motivated technique that yields clustering-like behavior is the technique used in self-organizing maps (Kohonen, 1988) where competition between different feature neurons coupled with Hebbian plasticity fosters the formation of dissimilar and informative features. These methods share the limitation that features are not learned in a label-guided manner. Auto-encoding-based methods learn features locally by minimizing the error in reconstructing one layer using the activity of the layer above (Bengio, 2014). Predictive coding methods attempts to minimize a similar reconstruction loss (Rao and Ballard, 1999). The unsupervised auto-encoding loss can be augmented by a supervised label-dependent loss to learn features that are label-guided and can thus be used to discriminate between different classes (Rasmus et al., 2015; Valpola, 2015). The supervised label-dependent error, however, is non-local.

In Baldi et al. (2016), Lillicrap et al. (2016), Nøkland (2016), and Neftci et al. (2017), the backpropagation scheme is modified to use random fixed weights in the backward path. This relaxes one of the biologically unrealistic requirements of backpropagation which is weight symmetry between the forward and backward pathways. Errors are still non-local, however, as they are generated by the top layer. A learning mechanism that is able to generate error signals locally is the synthetic gradients mechanism (Jaderberg et al., 2016; Czarnecki et al., 2017) in which errors are generated by dedicated error modules in each layer based only on the layer's activity and the label. The parameters of these dedicated error modules are themselves updated based on errors arriving from higher layers in order to make the error modules better predictors of the true, globally-derived, error signal. Our approach generates errors in a different manner through the use of a local classifier, and each layer receives no error information from the layer above.

## 3. Methods

**Figure 1:**
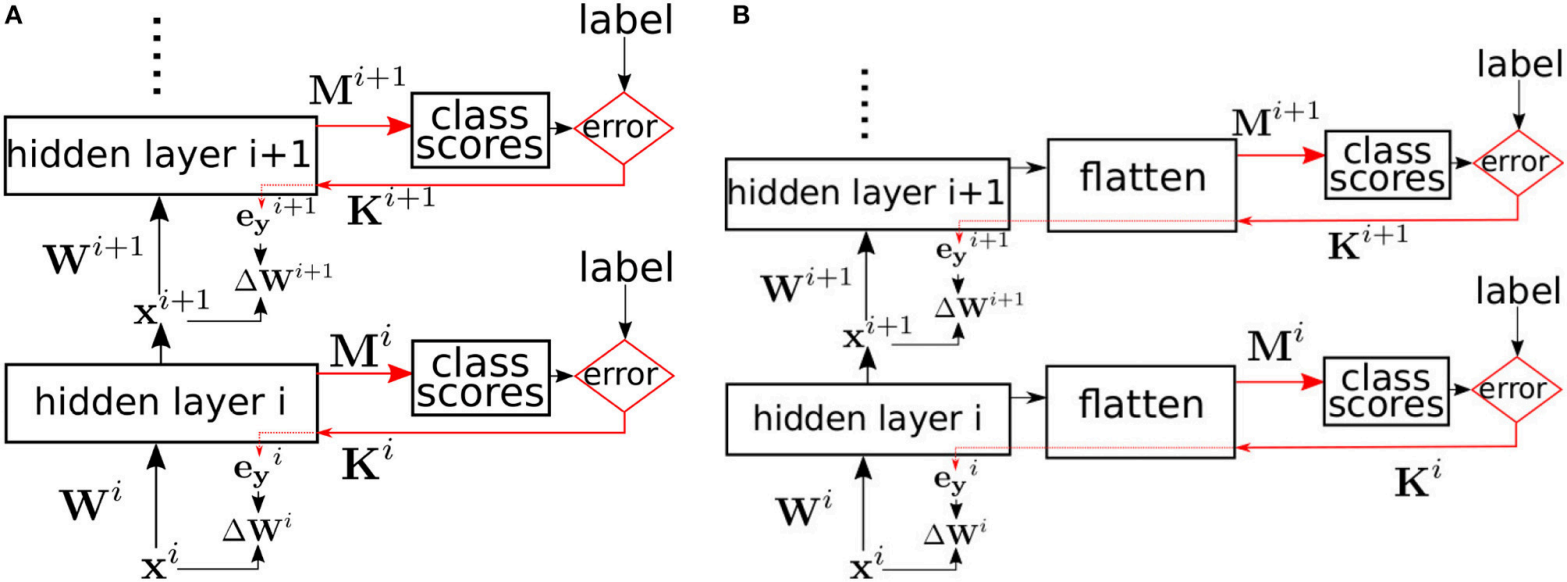
**(A)** Supervised learning in a multi-layer network using local errors. Biases are omitted for clarity. Red arrows indicate the error pathways. Hidden layer *i* is trained using local errors generated by a classifier with random fixed weights **M**^*i*^. The errors are randomly projected back using the fixed random matrix **K**^*i*^, and multiplied element-wise with the layer's activation derivative to yield the error signal ${{\text{e}}_{\text{y}}}^{i}$ which is then used to update the weights. **(B)** Same training method as **(A)** but applied to convolutional layers. The activation tensor of the convolutional layers is flattened to yield a 1D vector which is then used as an input to the local classifier.

We train multi-layer networks, with either convolutional or fully connected layers, based on local errors generated by random classifiers. Consider a fully connected *i*th hidden layer in a network whose activation vector is denoted by **y**^*i*^ ∈ *R*^*N*^ receiving an input **x**^*i*^ ∈ *R*^*M*^:

(1) $$\begin{array}{l} {\text{y}}^{i}=f\left({\text{W}}^{i}{\text{x}}^{i}+{\text{b}}^{i}\right) \end{array}$$

(2) $$\begin{array}{l} {\text{y}{\;}^{i}}^{\prime }=f^{\prime }\left({\text{W}}^{i}{\text{x}}^{i}+{\text{b}}^{i}\right) \end{array}$$

where **W**^*i*^ is the *N*×*M* weight matrix of layer *i* and **b**^*i*^ ∈ *R*^*N*^ is the bias vector, and *f* is the neuron's activation function, and *f*′ is the neuron's activation function derivative. In all the networks we train, we use Rectified Linear Units (ReLUs) (Nair and Hinton, 2010), i.e., *f*(*x*) = *max*(*x*, 0), with corresponding derivatives *f*′(*x*) = *H*(*x*) where *H*(·) is the Heaviside step function. f(x) = max(0,x) is a piecewise linear function. If the argument, x, is negative, then the function has a fixed output (zero) and its derivative is thus zero. If x is positive, then f is the identity function and its derivative is one. i.e., f'(x) = H(x) where H(x) is the Heaviside step function whose output is zero for negative x and one for positive x. We pre-define for this hidden layer a fixed random classifier matrix **M**^*i*^ which is a *C* × *N* matrix where *C* is the number of classification categories. The random matrix, **M**^*i*^, is used to convert the layer activation vector, **y**^*i*^, to a category score vector **s**^*i*^ ∈ *R*^*C*^ where **s**^*i*^ = **M**^*i*^**y**^*i*^. Since this is a supervised learning setting, the correct input category *t* is known during training, which allows the layer to generate a scalar loss or error signal, *E*(*t*, **s**^*i*^). *E* could be for example the cross-entropy loss or the square hinge loss. This error is then backpropagated in order to calculate the weight and bias updates, Δ**W**^*i*^ and Δ**b**^*i*^:

(3) $${\text{e}}_{\text{s}}{\;}^{i}=\frac{dE(t,{\text{s}}^{i})}{d{\text{s}}^{i}}$$

(4) $${{\text{e}}_{\text{y}}}^{i}={\text{K}}^{\text{i}}{\text{e}}_{\text{s}}^{\text{i}}⊙\text{y}i$$

(5) $$\Delta {\text{W}}^{i}=-\eta {\text{e}}_{\text{y}}{\;}^{i}\times {\text{X}}^{i}$$

(6) $$\Delta {\text{b}}^{i}=-\eta {\text{e}}_{\text{y}}{\;}^{i}$$

where ⊙ is the element-wise multiplication operator, × is the outer product operator, and η is the learning rate. **K**^*i*^ is the *N* × *C* matrix used to backpropagate the classifier error to the layer being trained. If we set **K**^*i*^ = **M**^*i*^*T*^^, then the weight and bias updates are executing exact gradient descent to minimize the random classifier error, *E*. In that case, training of each layer is equivalent to training a network with one hidden layer where only the hidden layer's input weights and biases are trainable, while the output weights, **M**^*i*^ are fixed. Equations 3–6 are then the standard backpropagation equations implementing gradient descent in order to minimize the local error. These equations were derived by applying the derivative chain rule in order to calculate the gradient of the error *E*(*t, s*^*i*^) with respect to the layer's weights and biases. The weights and biases are then updated to move in the negative gradient direction in order to minimize the error. With a small enough learning rate, gradient descent is guaranteed to converge to a local minimum of the error. The learning scheme is illustrated in Figure 1. Note that all random classifiers are always trained with the real labels to solve the actual learning task, whether it is MNIST or CIFAR10. When we say “random classifier,” we mean a classifier with random fixed weights, not that it solves a random task. The local random classifiers attain good accuracy because the layers feeding into them learn the right features that allow each particular random classifier to produce the correct label.

For convolutional layers, the learning scheme remains unchanged. The post-activation feature maps tensor is simply flattened to yield a 1D vector before multiplying by the random classifier matrix **M** as shown in Figure 1B. The flatten operation is just a reorganization of the activation tensor. We use an automatic differentiation (AD) package, PyTorch, to calculate the updates to a layer's weight in order to minimize the local classifier scalar cross-entropy loss. Convolutional layer kernels sweep over the input feature maps and are the same at different spatial positions. A convolutional layer can be thought of as a fully-connected layer, but with many weights tied together to reflect the spatial invariance of the convolution kernels. An AD package takes care of accumulating the gradient of each tied weight and summing these gradients to obtain the gradient of the underlying parameter.

### 3.1. Implementation details

In many experiments, we use dropout (Srivastava et al., 2014) to minimize overfitting. All incoming/outgoing weights to/from dropped neurons are not updated in the iteration in which the neuron is dropped. In custom hardware devices, dropout regularization can be implemented easily if we have access to a steady source of random bits. Uniformly distributed pseudo random numbers can be generated cheaply using a linear feedback shift register (LFSR) Klein (2013) as implemented in ref. Mostafa et al. (2017) on FPGAs. Generating new random numbers from LFSRs is very computationally cheap as it involves only few bit-wise XOR operations and no MAC operations. For example, in the scheme used in Cauwenberghs (1996), the number of flip flops needed to produce *N*_*b*_ random bits per cycle grows with $\sqrt{N_{b}}$. This scheme was adopted in Mostafa et al. (2017) that used 60 flip flops and 650 XOR gates to generate 320 random bits every clock cycle. In the training experiments reported in this paper, we did not use an explicit LFSR, but instead used a standard software-based random number generator. The end result is the same as if using an LFSR-based random number generator as both schemes can be used to generate uniformly distributed random numbers.

In some networks, we use batch normalization (Ioffe and Szegedy, 2015) before the layer's non-linearity. The layer's learnable parameters will then include a scaling factor (one for each neuron in a fully connected layer, or one for each feature map in a convolutional layer) that is also trained using local errors. Batch normalization is a common technique used in deep neural networks in order to normalize the input statistics to each neuron to have zero mean and unit variance. Batch normalization operates by calculating the mean and variance statistics of the input to each neuron (either independently in each mini-batch or using an exponential moving average across the whole training set). These statistics are then used to normalize the input to each neuron. Experimentally, batch normalization greatly accelerates learning by ensuring the inputs to the neurons do not take extreme values, thereby making learning more stable. During inference, batch normalization incurs a minor extra computational overhead of one multiplication and one division per neuron.

For a fully connected layer, the input to the local classifier is taken after the dropout mask is applied (if dropout is used). For a convolutional layer, the input to the layer's local classifier is taken after pooling and after applying the dropout mask. In all experiments, we initialize the fixed random classifier weights from a zero-mean distribution whose standard deviation depends on the fan-out of the neurons in the source layer, *n*_*out*_, and the fan-in of the neurons in the target layer, *n*_*in*_, according to

(7) $$\begin{array}{l} std=\sqrt{\frac{2}{n_{in}+n_{out}}} \end{array}$$

We experimented with both uniform distributions and Gaussian distributions when choosing the random weights of the local classifier.

We compare our approach to the feedback alignment training method (Lillicrap et al., 2016) in which random fixed weights are used to backpropagate the error layer-by-layer from the top layer. The layer's activation derivative is still used when backpropagating errors. In the presence of max-pooling layers, errors only backpropagate through the winner(max) neuron in each pooling window. When using feedback alignment training in the presence of dropout, a neuron that is dropped during the forward pass is also dropped during the backward pass. When using convolutional layers, we use fixed random filters that we convolve with the errors of one convolutional layer to yield the errors at the outputs of the previous/lower convolutional layer. We also use batch normalization when training using feedback alignment. The extra scaling parameters introduced by batch normalization are trained using the randomly backpropagated errors arriving at the batch-normalized layer's output.

The local error learning mechanism we describe in this paper can either be applied in a mini-batch setting or in an online learning setting where data is presented sample by sample (mini-batch size of 1). In the training experiments we present, we use mini-batch training in order to accelerate learning on the GPUs we use. This decision was purely for GPU performance reasons in order to complete training in a reasonable amount of time. Evidence points that using smaller mini-batch sizes down to a mini-batch size of 1 will improve our results by improving the network stability (Masters and Luschi, 2018) and generalization ability (Keskar et al., 2016). All experiments in this paper were carried out using Pytorch, and all parameters were optimized using stochastic gradient descent with Nesterov accelerated gradient (NAG) (Nesterov, 1983; Ruder, 2016). We use a momentum of 0.9 and a starting learning rate of 0.1. We train for 100 epochs and reduce the learning rate by a factor of 5 every 25 epochs. The full code used to run all experiments is available under https://gitlab.com/hesham-mostafa/learning-using-local-erros.git.

## 4. Hardware implications of learning using local errors

Standard learning techniques based on backpropagating errors through the whole network require the hardware executing the learning algorithm to store the activation values and activation derivatives of all network layers in order to calculate weight updates and backpropagate errors once errors are available from the top layer. This imposes several communication and memory access overheads if learning is executed on hardware whose memory can not accommodate all the network weights and activations. For large scale convolutional networks, this practically includes all CPU and GPU devices where on-chip memory is limited to few tens of MBytes, while state of the art deep convolutional networks typically require several hundred MBytes to several GBytes in order to store the network weights and mini-batch activations (Rhu et al., 2016). Data thus has to be continuously shuttled between the compute device and external memory. This is the case even in custom accelerators developed to accelerate just the inference (feed-forward) phase (Himavathi et al., 2007; Cavigelli et al., 2015; Ardakani et al., 2017; Chen et al., 2016; Han et al., 2016; Aimar et al., 2017; Jouppi et al., 2017), where a complete forward pass through a large-scale convolutional network can not be executed completely on the accelerator without having to access external memory to store intermediate activations and to load weights.

Energy needed to drive off-chip traffic from/to external memory as well as memory read/write energy often contribute significantly to the overall energy consumption of a data intensive task such as deep learning (Lefurgy et al., 2003; Vogelsang, 2010; Chen et al., 2016). Reducing memory traffic can thus have significant impact on the overall energy consumption of the learning hardware. Moreover, since communication with external memory is typically done through the chip border (in the absence of 3D stacking), external memory bandwidth scales linearly with chip size, whereas compute resources and internal memory scale quadratically with chip size. Minimizing external memory access is thus key to improving performance and power efficiency. In this section, we analyze the savings in memory traffic volume obtained using the learning approach based on local errors that we propose in this paper. Note that the random weights used in the local classifiers have virtually zero memory overhead as they can be generated on the fly using a PRNG.

Consider a neural network with *L* layers. *P*^*i*^ and *A*^*i*^ are the parameters and the mini-batch activations of layer *i*, respectively. |*P*^*i*^| and |*A*^*i*^| are the number of elements in *P*^*i*^ and *A*^*i*^. A neuron in layer *i* has a fanout of *R*^*i*^, i.e., a neuron in layer *i* projects to *R*^*i*^ neurons in layer *i* + 1. In convolutional layers, we ignore any border effects which might cause the neurons at the borders of the feature maps to project to fewer neurons than neurons away from the borders. We divide the training data set into *N*_*b*_ mini-batches and train the network for *N*_*e*_ epochs. Each weight and each neuron activation takes up one memory word (which we assume is 32 bits).

**Figure 2:**
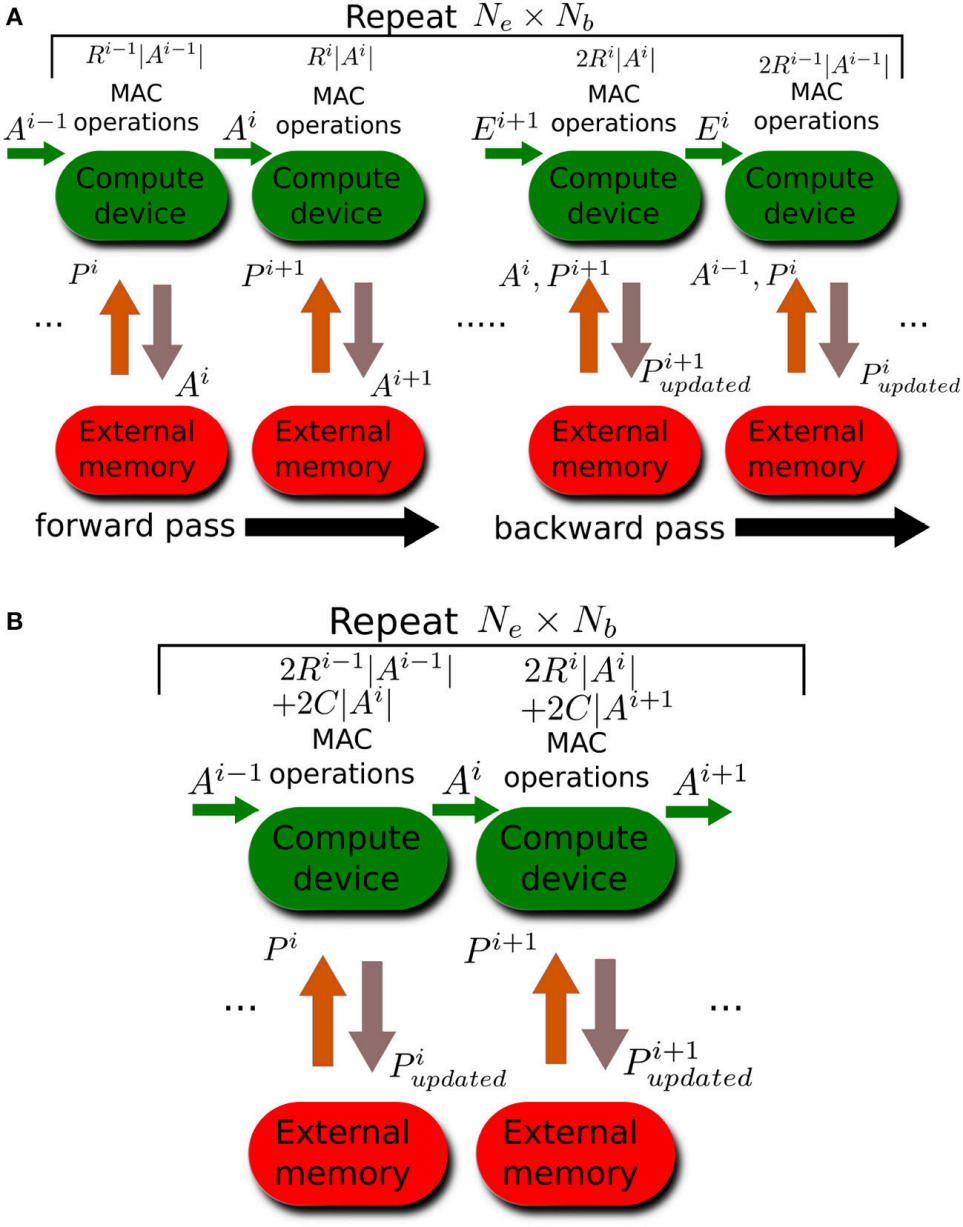
Memory traffic and number of MAC operations for different training methods. Arrows between compute device and external memory indicate memory traffic while green arrows indicate data buffered and reused by the compute device. Each computation stage is executed a number of times given by the enclosing repeat block. **(A)** Standard backpropagation learning. **(B)** Training all layers simultaneously using local errors. Note that there is no backward pass as weights are updated during the forward pass.

Figure 2A illustrates the data traffic and the number of MAC operations needed during standard backpropagation training. The data traffic in Figure 2A assumes the compute device has enough on-board memory to buffer the output activations of one layer in order to use these activations to calculate the next layer's activation. We also assume the compute device does not need the parameters of any layer to be streamed in more than once during each forward pass and during each backward pass. These assumptions would hold true if the accelerator has at least $\underset{i}{\max}\left(\left|P^{i}|+N_{b}|A^{i}\right|\right)$ words of on-board memory. For layer *i*, the compute device needs to read in the layer's parameters *P*^*i*^. It will calculate the layer's activations, *A*^*i*^. It will then have to stream out these activations so that they can be used to update the weights during the backward pass. The number of MAC operations needed to calculate the activation of layer *i* is *R*^*i*−1^|*A*^*i*−1^| which is the product of the number of neurons in layer *i* − 1 and the number of each neuron's output connections. During the backward pass, the compute device buffers the back-propagated errors of one layer and uses them to calculate the errors at the preceding layer. *R*^*i*^|*A*^*i*^| MAC operations are needed to calculate the weight updates for *P*^*i*+1^. An additional *R*^*i*^|*A*^*i*^| MAC operations are needed to backpropagate the errors from layer *i* + 1 to layer *i*. We ignore the special case of the input layer where errors do not need to be backpropagated. We also ignore the MAC operations needed to calculate the error at the top layer.

Figure 2B illustrates the case when learning is done using errors generated by random local classifiers. As in standard backpropagation, *R*^*i*−1^|*A*^*i*−1^| MAC operations are needed to calculate the activations of layer *i*. To calculate the local classifier output, *C*|*A*^*i*^| MAC operations are needed where *C* is the number of classification classes. Note that the random classifier weights can be generated on the fly using a PRNG, and thus only require the PRNG seed (whose size can be 32 bits for 32-bit weights) to be stored. To backpropagate the local classifier error to obtain the error at layer *i*, an additional *C*|*A*^*i*^| MAC operations are needed and *R*^*i*−1^|*A*^*i*−1^| MAC operations are needed to update the parameters of layer *i*, *P*^*i*^, based on the layer's error. Note that the crucial difference between standard backpropagation and learning using local errors is that the latter does not need to stream out the activations since these activations are immediately used to update the layer's parameters based on the locally generated errors and do not need to be remembered for later. Moreover, learning using local errors only needs to read in the parameters once (not twice as in standard backpropagation) as the parameters are updated immediately using local errors as soon as they are used to calculate the activations of the current layer.

Table 1 summarizes the number of MAC operations and the memory read/write volume required by the two training methods. Learning using local errors has a decisive advantage when it comes to memory traffic as it requires drastically less read and write operations compared to standard backpropagation. The reduction in the number of MAC operations is less unequivocal as it depends on the number of classification classes, *C*, and the fanout of the neurons in the network, *R*^*i*^. Learning using local error reduces the MAC operations count if $L\times C<0.5\underset{i}{\sum }R^{i}$. This condition is easily satisfied when the number of classes is small and it was satisfied by all the networks presented in this paper.

**Table 1:** Memory traffic and number of MAC operations for different learning methods.

**Training method**	**Memory read (words)**	**Memory write (words)**	**MAC operations**
Standard backpropagation	$N_{e}N_{b}\underset{i}{\sum }\left(2|P^{i}|+|A^{i}|\right)$	$N_{e}N_{b}\underset{i}{\sum }\left(\left|P^{i}|+|A^{i}\right|\right)$	$N_{e}N_{b}\underset{i}{\sum }3R^{i}|A^{i}|$
Learning using local errors	$N_{e}\underset{i}{\sum }|P^{i}|$	$N_{e}\underset{i}{\sum }|P^{i}|$	$N_{e}N_{b}\underset{i}{\sum }\left(2R^{i}+2C\right)|A^{i}|$

*N_e_ and N_b_ are the numbers of epochs and the number of minibatches in an epoch, respectively. P^i^ is the layer's parameter tensor and A^i^ is the layer's mini-batch activations. R^i^ is the fan-out of each neuron in layer i*.

## 5. Results

### 5.1. MNIST

**Figure 3:**
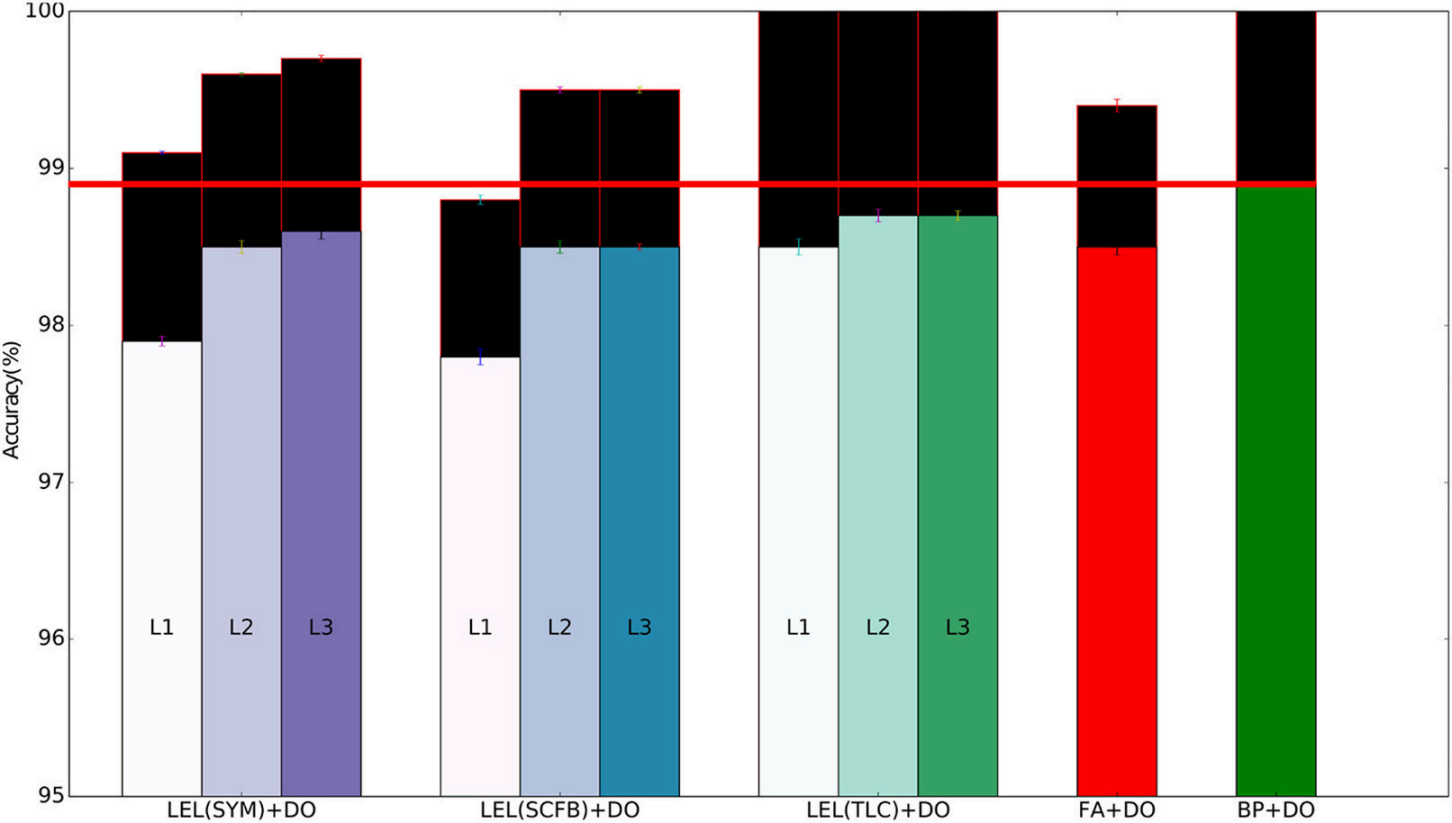
Training and test set accuracy on the MNIST dataset using a 3-layer fully-connected network. Results are shown for 5 training methods. When training using local errors, the accuracies of the local classifiers in all layers are shown. LEL, Local Error Learning; SYM, Symmetric feedback weights; SCFB, Sign-concordant feedback weights; TLC, Trainable local classifier; DO, Dropout; FA, Feedback alignment; BP, Backpropagation. Colored bars indicate test set accuracy. The black bars above the colored bars indicate the accuracy on the training set which is always larger than test-set accuracy. The height of the black bars thus indicate the generalization gap, or the difference between training set accuracy and test set accuracy. The horizontal red line indicates the test accuracy when the network was trained using standard backpropagation.

We first validate the performance of our training approach on the MNIST hand-written digit recognition task. We used the standard split of 50,000/10,000/10,000 examples for training/validation/testing respectively. The validation set was added to the training set after choosing the hyper-parameters. We use a network with 3 fully connected hidden layers with 1,000 neurons per layer and train the weights in the entire network using local errors. As a baseline, we also train a 3-hidden layers network using standard backpropagation where each hidden layer also has 1,000 neurons. We experimented with using dropout to reduce overfitting. We first used fixed symmetric random weights in the forward and backward pathways in the local error loops, i.e., **K**^*i*^ = **M**^*i*^*T*^^ in all layers. The local classifier errors improve for the second and third hidden layers compared to the first hidden layer, implying that the network is able to make use of depth to obtain better accuracy. The local classifier errors in the second and third layers are similar implying that the network is unable to make use of the increased depth beyond two hidden layers for this simple dataset. When training using local errors, we also ran experiments where the local classifier weights were trainable parameters, i.e., the local classifier weights, **M**^*i*^, are trainable. When training local classifier weights, these weights are updated during each training iteration in order to minimize the error of the local classifier using standard gradient descent, i.e., the gradient of the local classifier error with respect to these weights was calculated and the weights were updated to move in the negative gradient direction to minimize the local error.This had minimal effect on accuracy as shown in Table 2. We experimented with changing the distribution of the local random classifier weights from a uniform distribution to a Gaussian distribution. As shown in Table 2, performance was unchanged. Figure 3 summarizes the main findings from Table 2.

**Table 2:** MNIST final train and test accuracy after 100 training epochs.

		**Layer 1**	**Layer 2**	**Layer 3**
LEL(SYM)	Train	99.8 ± 0.00%	100.0 ± 0.00%	100.0 ± 0.00%
	Test	97.8 ± 0.03%	98.2 ± 0.07%	98.2 ± 0.05%
LEL(SYM) + DO	Train	99.1 ± 0.01%	99.6 ± 0.01%	99.7 ± 0.02%
	Test	97.9 ± 0.03%	98.5 ± 0.04%	98.6 ± 0.05%
LEL(SYM) + DO/GI	Train	99.0 ± 0.02%	99.6 ± 0.02%	99.6 ± 0.02%
	Test	98.0 ± 0.06%	98.5 ± 0.05%	98.6 ± 0.05%
LEL(SCFB) + DO	Train	98.8 ± 0.03%	99.5 ± 0.02%	99.5 ± 0.02%
	Test	97.8 ± 0.05%	98.5 ± 0.04%	98.5 ± 0.02%
LEL(TLC) + DO	Train	100.0 ± 0.00%	100.0 ± 0.00%	100.0 ± 0.00%
	Test	98.5 ± 0.05%	98.7 ± 0.04%	98.7 ± 0.03%
LEL(TLC)	Train	100.0 ± 0.00%	100.0 ± 0.00%	100.0 ± 0.00%
	Test	98.2 ± 0.06%	98.4 ± 0.04%	98.4 ± 0.03%
FA + DO	Train	–	–	99.4 ± 0.04%
	Test	–	–	98.5 ± 0.05%
BP	Train	–	–	100.0 ± 0.0%
	Test	–	–	98.8 ± 0.04%
BP + DO	Train	–	–	99.9 ± 0.01%
	Test	–	–	98.9 ± 0.01%

*When learning using local errors, the local classifier errors in all layers are reported. Mean and standard deviation from 4 runs. LEL, Local error learning; SYM, Symmetric feedback weights; SCFB, Sign-concordant feedback weights; TLC, Trainable local classifier; DO, Dropout; FA, Feedback alignment; BP, Backpropagation; GI, Gaussian initialization of local classifier weights. For local error learning, local classifier weights were initialized from a uniform distribution, except for cases with marker GI*.

Next, to lessen concerns about the biological implausibility of exact symmetry in feedforward and feedback weights, we relaxed the weight symmetry requirement in the local error loops and initialized the error feedback weights **K**^*i*^ randomly and independently of **M**^*i*^, except we then modified the sign of the weights in **K**^*i*^ so that *sign*(**K**^*i*^) = *sign*(**M**^*i*^*T*^^). The signs of the feedback weights in the local error loops thus match the signs of the feedforward weights (both are fixed and have independent magnitudes). This is the 'sign-concordant feedback weights' case in Table 2. Generating two random number streams with sign-concordant weights (i.e., corresponding numbers in the two streams have the same sign) can be achieved on hardware by running two LFSRs in parallel and using the sign of one LFSR to set the sign of the numbers generated by the other LFSR. Corresponding numbers in the two random streams would thus have the same sign but independent (and random) magnitudes. There was minimal impact on performance compared to symmetric feedforward and feedback local classifier weights. When we relax the symmetry requirement further and choose **K**^*i*^ to be random and completely independent of **M**^*i*^, the network failed to learn and error rates stayed at near-chance level. We also experimented with training based on feedback alignment where errors from the top layer are backpropagated using random fixed weights. The network's performance using feedback alignment is comparable to performance using local errors as shown in Table 2.

It is important to note that in feedback alignment, the feedforward weights eventually “align” with the random weights used to backpropagate errors (Lillicrap et al., 2016) enabling the network to learn. When learning using random fixed local classifiers, and if we choose random error feedback weights, the classifier weights are fixed and thus can not align with the random weights used in the one-step backpropagation. Reliable error information, however, can still reach the layer being trained if the signs of the random backpropagation weights, **K**^*i*^, match the signs of the fixed local classifier weights **M**^*i*^. This is in-line with previous investigations into the importance of weight symmetry in backpropagation that argue for the importance of sign-concordance between forward and backward weights (Liao et al., 2016).

### 5.2. CIFAR10

We trained a convolutional network with three convolutional layers followed by two fully connected layers on the CIFAR10 dataset. We used a similar network as Srivastava et al. (2014). The convolutional layers used a 5 × 5 kernel, a stride of 1, and had 96, 128, and 256 feature maps going from the bottom upwards. Max-pooling with a pooling window of 3 × 3 and stride 2 was applied after each convolutional layer. The two fully connected layers on top had 2, 048 neurons each. All layers were batch-normalized and in experiments where dropout was used, dropout was applied after the input layer, after each max-pooling layer, and after each fully connected layer.

**Figure 4:**
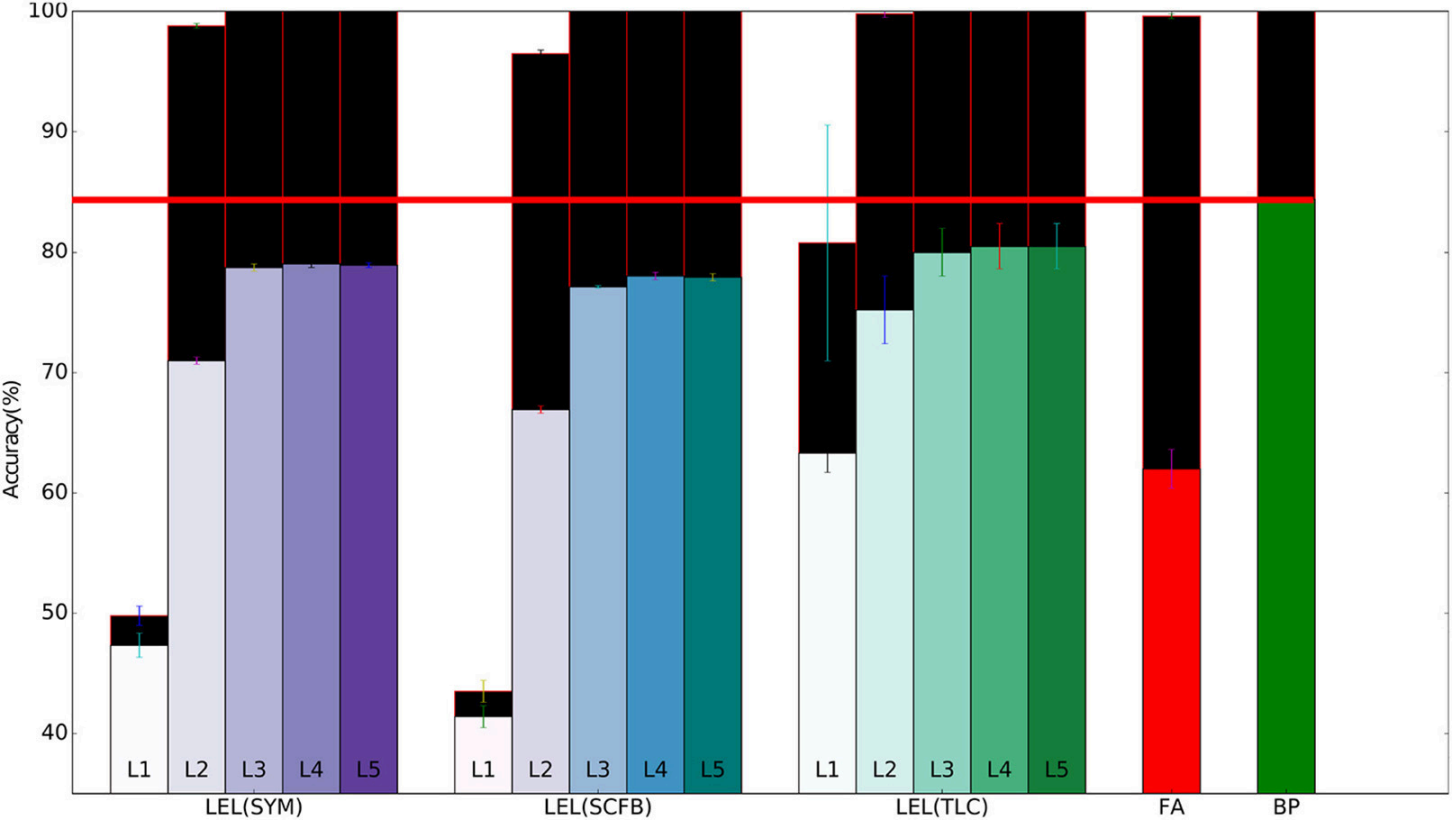
Training and test set accuracy on the CIFAR10 dataset using a 5-layer network. Results are shown for 5 training methods. When training using local errors, the accuracies of the local classifiers in all layers are shown. LEL, Local Error Learning; SYM, Symmetric feedback weights; SCFB, Sign-concordant feedback weights; TLC, Trainable local classifier; FA, Feedback alignment; BP, Backpropagation. Colored bars indicate test set accuracy. The black bars above the colored bars indicate the accuracy on the training set which is always larger than test-set accuracy. The height of the black bars thus indicate the generalization gap, or the difference between training set accuracy and test set accuracy. The horizontal red line indicates the test accuracy when the network was trained using standard backpropagation.

The 32×32×3 CIFAR10 color images were pre-processed by subtracting the per-pixel mean and dividing by the per-pixel standard deviation. The training set of 50,000 images was used for training/validation and we report errors on the 10,000 images test set. Unlike the MNIST dataset, standard backpropagation significantly outperforms training using local errors as shown in Table 3. Performance of local error learning deteriorates slightly when using sign-concordant local feedback weights instead of symmetric local feedback weights. The local classifiers are not part of the network feedforward path. Their role is to provide error signals during training and provide a pathway to obtain classification decisions from intermediate layers. By making these classifiers learnable, training can now adjust both the network weights and classifier weights to minimize the local classifier errors. The increased number of trainable parameters thus leads to better performance as shown in Table 3. We carried out experiments in which the first one or two convolutional layers were fixed, i.e., their weights were fixed at at their random initialization points. As shown in Table 3, performance suffers indicating that local learning at early layers is important in order to allow them to learn useful features that can then be used by higher layers. Note that even though we fixed these initial layers, their local classifier error is above chance since the batch normalization parameters for these layers are still learnable allowing the performance of the local classifiers to go above chance. Training the local classifier leads to slightly improved performance. Unlike the MNIST network, for this deeper network, training using feedback alignment leads to significantly worse performance compared to learning using local errors. Figure 4 summarizes the main findings from Table 3.

**Table 3:** CIFAR10 final train and test accuracy after 100 training epochs on a 5-layer network.

		**conv1**	**conv2**	**conv3**	**FC1**	**FC2**
LEL(SYM)	Train	49.8 ± 0.8%	98.8 ± 0.2%	100.0 ± 0.0%	100.0 ± 0.0%	100.0 ± 0.0%
	Test	47.3 ± 1.0%	71.0 ± 0.3%	78.7 ± 0.3%	79.0 ± 0.3%	78.9 ± 0.2%
LEL(SYM)-Fix 1	Train	22.0 ± 0.8%	91.2 ± 1.0%	100.0 ± 0.0%	100.0 ± 0.0%	100.0 ± 0.0%
	Test	22.5 ± 0.7%	66.5 ± 0.5%	75.2 ± 0.2%	75.5 ± 0.1%	75.4 ± 0.1%
LEL(SYM)-Fix 2	Train	22.7 ± 1.0%	27.0 ± 1.3%	99.2 ± 0.1%	100.0 ± 0.0%	100.0 ± 0.0%
	Test	23.1 ± 0.9%	27.4 ± 1.3%	68.2 ± 0.2%	68.5 ± 0.1%	68.3 ± 0.2%
LEL(SYM) + DO	Train	46.3 ± 0.7%	75.2 ± 0.9%	87.2 ± 0.7%	88.8 ± 0.6%	89.1 ± 0.6%
	Test	44.8 ± 0.9%	67.8 ± 0.8%	76.4 ± 0.5%	78.1 ± 0.5%	78.1 ± 0.4%
LEL(SYM)/GI	Train	50.0 ± 0.4%	98.7 ± 0.3%	100.0 ± 0.0%	100.0 ± 0.0%	100.0 ± 0.0%
	Test	47.5 ± 0.3%	71.1 ± 0.3%	78.6 ± 0.2%	78.8 ± 0.3%	78.6 ± 0.3%
LEL(SYM)+DO/GI	Train	46.6 ± 1.1%	75.6 ± 0.7%	87.4 ± 0.5%	89.0 ± 0.4%	89.2 ± 0.4%
	Test	44.8 ± 1.3%	68.3 ± 0.5%	76.6 ± 0.5%	78.5 ± 0.2%	78.3 ± 0.4%
LEL(SCFB)+DO	Train	39.8 ± 1.0%	72.4 ± 0.9%	85.4 ± 0.8%	87.7 ± 0.6%	88.1 ± 0.7%
	Test	38.5 ± 1.2%	65.4 ± 0.5%	75.6 ± 0.6%	78.1 ± 0.5%	78.3 ± 0.6%
LEL(SCFB)	Train	43.5 ± 0.9%	96.5 ± 0.3%	100.0 ± 0.0%	100.0 ± 0.0%	100.0 ± 0.0%
	Test	41.4 ± 0.9%	66.9 ± 0.3%	77.1 ± 0.1%	78.0 ± 0.3%	77.9 ± 0.3%
LEL(TLC)+DO	Train	70.9 ± 2.1%	86.5 ± 2.5%	97.2 ± 1.2%	98.2 ± 0.7%	97.6 ± 1.0%
	Test	62.4 ± 0.7%	73.8 ± 0.8%	78.0 ± 1.2%	79.2 ± 1.1%	79.1 ± 1.2%
LEL(TLC)	Train	80.8 ± 9.8%	99.8 ± 0.3%	100.0 ± 0.0%	100.0 ± 0.0%	100.0 ± 0.0%
	Test	63.3 ± 1.6%	75.2 ± 2.8%	80.0 ± 2.0%	80.5 ± 1.9%	80.5 ± 1.9%
FA	Train	–	–	–	–	99.6 ± 0.2%
	Test	–	–	–	–	62.0 ± 1.6%
BP	Train	–	–	–	–	100.0 ± 0.0%
	Test	–	–	–		84.4 ± 0.1%
BP + DO	Train	–	–	–	–	99.6 ± 0.06%
	Test	–	–	–	–	84.0 ± 0.4%

*When learning using local errors, the local classifier errors in all layers are reported. Mean and standard deviation from 4 runs. LEL, Local error learning; SYM, Symmetric feedback weights; SCFB, Sign-concordant feedback weights; TLC, Trainable local classifier; DO, Dropout; FA, Feedback alignment; BP, Backpropagation; GI, Gaussian initialization of local classifier weights. For local error learning, local classifier weights were initialized from a uniform distribution, except for cases where GI is indicated. “Fix n” means the parameters of the first n convolutional layers in the network were random and non-trainable*.

**Figure 5:**
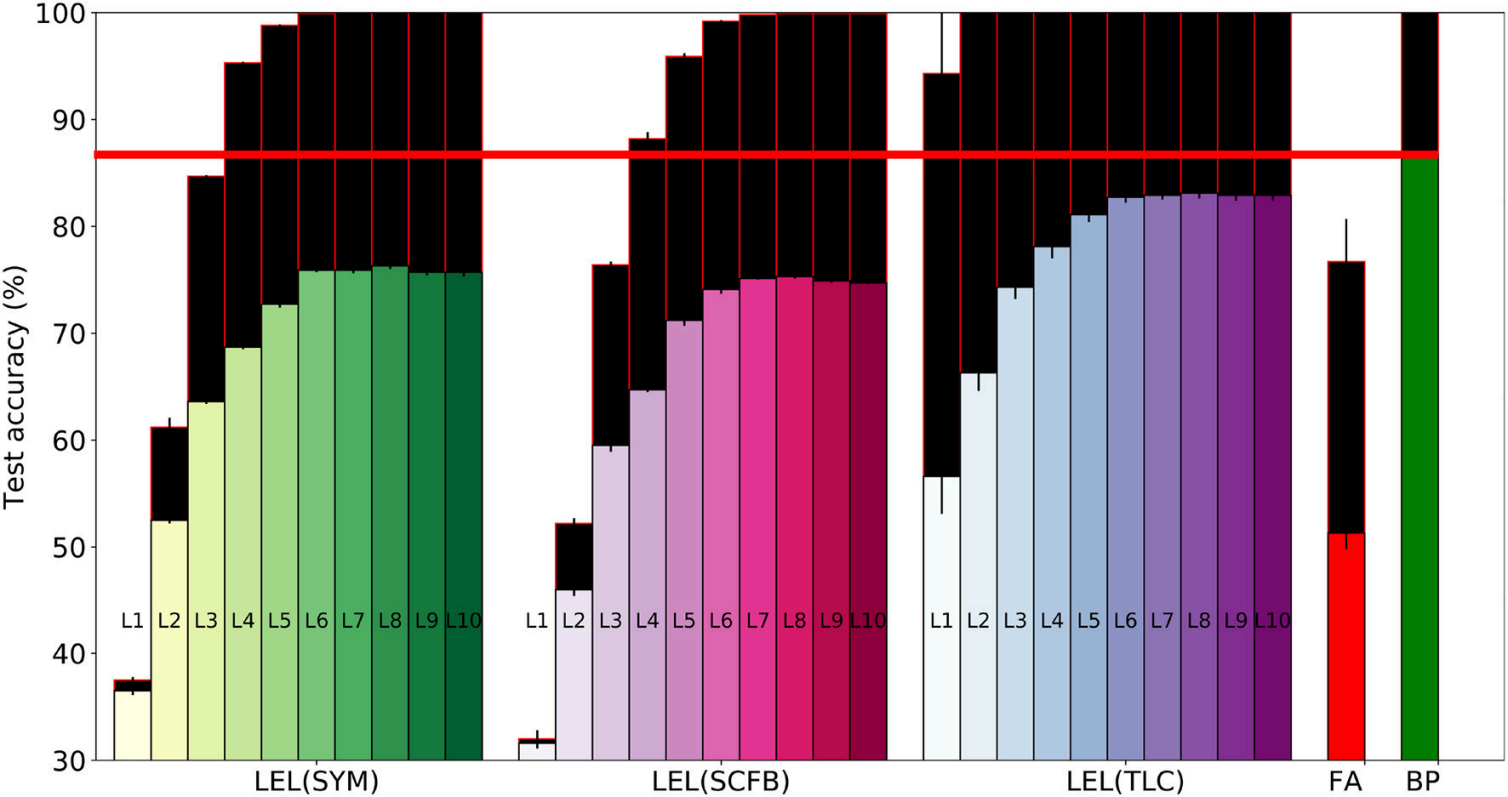
Training and test set accuracy on the CIFAR10 dataset using a 10-layer convolutional network. Results are shown for 5 training methods. When training using local errors, the accuracies of the local classifiers in all layers are shown. LEL, Local error learning; SYM, Symmetric feedback weights; SCFB, Sign-concordant feedback weights; TLC, Trainable local classifier; FA, Feedback alignment; BP, Backpropagation. Colored bars indicate test set accuracy. The black bars above the colored bars indicate the accuracy on the training set which is always larger than test-set accuracy. The height of the black bars thus indicate the generalization gap, or the difference between training set accuracy and test set accuracy. The horizontal red line indicates the test accuracy when the network was trained using standard backpropagation.

To see how learning using local errors scales, we experimented with a 10-layer deep convolutional network. The network has the layer stack: C64-C64-MP-C128-C128-MP-C256-C256-MP-C512-C512-MP-C512-C512-MP, where Cx indicates a convolutional layer with x feature maps and 3x3 kernels, and MP indicates a max-pooling layer. We show the learning performance for the first 5 convolutional layers in Table 4 and for the last 5 convolutional layers in Table 5. Learning using local errors still lags behind standard backpropagation but it still significantly outperforms learning using feedback alignment. Making the local classifiers trainable significantly improves performance in this network. Using Gaussian initialization for the fixed random classifier weights has minimal effect on performance. Figure 5 summarizes the main findings from Tables 4, 5.

**Table 4:** CIFAR10 final train and test accuracy after 100 training epochs on the first 5 layers of 10-layer network.

		**conv1**	**conv2**	**conv3**	**conv4**	**conv5**
LEL(SYM)	Train	37.5 ± 0.3%	61.2 ± 0.9%	84.7 ± 0.1%	95.3 ± 0.1%	98.8 ± 0.1%
	Test	36.5 ± 0.4%	52.5 ± 0.3%	63.6 ± 0.2%	68.7 ± 0.2%	72.7 ± 0.3%
LEL(SYM)-Fix 1	Train	19.9 ± 1.1%	54.6 ± 1.9%	85.8 ± 1.2%	97.2 ± 0.4%	99.5 ± 0.1%
	Test	20.0 ± 0.9%	48.4 ± 1.1%	62.2 ± 0.2%	67.9 ± 0.6%	71.9 ± 0.4%
LEL(SYM)-Fix 2	Train	19.8 ± 1.0%	20.3 ± 0.9%	72.9 ± 1.9%	96.7 ± 0.5%	99.7 ± 0.1%
	Test	19.9 ± 1.0%	20.7 ± 1.4%	56.7 ± 0.6%	63.5 ± 0.7%	67.2 ± 0.6%
LEL(SYM) + DO	Train	37.0 ± 0.4%	49.2 ± 2.5%	63.0 ± 1.2%	65.8 ± 1.1%	66.6 ± 1.8%
	Test	36.4 ± 0.5%	47.1 ± 2.3%	57.3 ± 1.2%	61.9 ± 0.9%	60.7 ± 1.6%
LEL(SYM)/GI	Train	36.9 ± 0.4%	60.4 ± 0.8%	84.4 ± 0.3%	95.3 ± 0.2%	98.8 ± 0.1%
	Test	36.1 ± 0.6%	52.4 ± 0.7%	63.8 ± 0.6%	69.1 ± 0.3%	73.2 ± 0.5%
LEL(SYM)+DO/GI	Train	37.5 ± 0.2%	49.3 ± 1.2%	61.7 ± 2.9%	64.3 ± 2.3%	65.7 ± 4.8%
	Test	36.6 ± 0.5%	47.4 ± 0.9%	56.5 ± 2.1%	60.9 ± 1.9%	60.1 ± 4.2%
LEL(SCFB)+DO	Train	32.6 ± 0.8%	44.1 ± 0.5%	57.1 ± 3.9%	59.9 ± 4.1%	60.0 ± 7.0%
	Test	31.9 ± 1.2%	42.5 ± 0.7%	52.8 ± 3.3%	56.9 ± 3.7%	55.5 ± 6.1%
LEL(SCFB)	Train	32.0 ± 0.8%	52.2 ± 0.5%	76.4 ± 0.3%	88.2 ± 0.6%	95.9 ± 0.3%
	Test	31.6 ± 0.5%	46.0 ± 0.6%	59.5 ± 0.6%	64.7 ± 0.2%	71.2 ± 0.5%
LEL(TLC)+DO	Train	99.9 ± 0.1%	79.0 ± 3.0%	94.7 ± 3.0%	89.0 ± 2.4%	96.3 ± 1.9%
	Test	58.8 ± 1.8%	66.0 ± 1.1%	71.0 ± 1.9%	75.7 ± 1.5%	77.9 ± 1.6%
LEL(TLC)	Train	94.3 ± 8.5%	100.0 ± 0.0%	100.0 ± 0.0%	100.0 ± 0.0%	100.0 ± 0.0%
	Test	56.6 ± 3.5%	66.3 ± 1.7%	74.3 ± 1.1%	78.1 ± 1.1%	81.1 ± 0.7%

*When learning using local errors, the local classifier errors in all layers are reported. Mean and standard deviation from 4 runs. LEL, Local error learning; SYM, Symmetric feedback weights; SCFB, Sign-concordant feedback weights; TLC, Trainable local classifier; DO, Dropout; FA, Feedback alignment; BP, Backpropagation; GI, Gaussian initialization of local classifier weights. For local error learning, local classifier weights were initialized from a uniform distribution, except for cases where GI is indicated. “Fix n” means the parameters of the first n convolutional layers in the network were random and non-trainable*.

**Table 5:** CIFAR10 final train and test accuracy after 100 training epochs on the last 5 layers of 10-layer network.

		**conv6**	**conv7**	**conv8**	**conv9**	**conv10**
LEL(SYM)	Train	99.9 ± 0.0%	100.0 ± 0.0%	100.0 ± 0.0%	100.0 ± 0.0%	100.0 ± 0.0%
	Test	75.9 ± 0.2%	75.9 ± 0.3%	76.3 ± 0.3%	75.7 ± 0.3%	75.7 ± 0.4%
LEL(SYM)-Fix 1	Train	100.0 ± 0.0%	100.0 ± 0.0%	100.0 ± 0.0%	100.0 ± 0.0%	100.0 ± 0.0%
	Test	75.3 ± 0.1%	75.2 ± 0.2%	75.6 ± 0.2%	75.0 ± 0.2%	74.9 ± 0.2%
LEL(SYM)-Fix 2	Train	100.0 ± 0.0%	100.0 ± 0.0%	100.0 ± 0.0%	100.0 ± 0.0%	100.0 ± 0.0%
	Test	71.0 ± 1.0%	70.8 ± 0.8%	71.2 ± 0.8%	70.6 ± 0.8%	70.5 ± 0.7%
LEL(SYM) + DO	Train	66.2 ± 2.4%	69.9 ± 2.4%	70.5 ± 2.2%	67.8 ± 2.2%	67.9 ± 2.3%
	Test	61.5 ± 2.1%	63.2 ± 2.1%	64.2 ± 1.9%	62.7 ± 1.9%	62.8 ± 2.0%
LEL(SYM)/GI	Train	99.9 ± 0.0%	100.0 ± 0.0%	100.0 ± 0.0%	100.0 ± 0.0%	100.0 ± 0.0%
	Test	76.0 ± 0.3%	76.1 ± 0.5%	76.2 ± 0.5%	75.7 ± 0.6%	75.6 ± 0.6%
LEL(SYM)+DO/GI	Train	66.4 ± 4.7%	70.0 ± 4.7%	69.6 ± 5.0%	66.9 ± 5.2%	66.9 ± 5.2%
	Test	61.4 ± 4.2%	63.1 ± 3.7%	63.1 ± 3.9%	61.6 ± 4.2%	61.6 ± 4.3%
LEL(SCFB)+DO	Train	60.1 ± 6.7%	63.2 ± 5.6%	63.8 ± 5.8%	61.7 ± 5.9%	61.8 ± 6.0%
	Test	56.2 ± 6.0%	57.8 ± 4.8%	58.5 ± 4.9%	57.4 ± 5.3%	57.6 ± 5.4%
LEL(SCFB)	Train	99.2 ± 0.1%	99.8 ± 0.0%	99.9 ± 0.0%	99.9 ± 0.0%	99.9 ± 0.0%
	Test	74.1 ± 0.4%	75.1 ± 0.1%	75.3 ± 0.2%	74.9 ± 0.2%	74.7 ± 0.1%
LEL(TLC)+DO	Train	93.2 ± 1.9%	97.0 ± 1.4%	96.0 ± 1.5%	94.5 ± 1.9%	94.5 ± 1.9%
	Test	79.4 ± 1.5%	80.5 ± 1.4%	80.9 ± 1.3%	80.8 ± 1.4%	80.8 ± 1.3%
LEL(TLC)	Train	100.0 ± 0.0%	100.0 ± 0.0%	100.0 ± 0.0%	100.0 ± 0.0%	100.0 ± 0.0%
	Test	82.7 ± 0.5%	82.9 ± 0.4%	83.1 ± 0.5%	82.9 ± 0.5%	82.9 ± 0.5%
FA	Train	–	–	–	–	76.7 ± 4.0%
	Test	–	–	–	–	51.3 ± 1.5%
BP	Train	–	–	–	–	100.0 ± 0.0%
	Test	–	–	–	–	86.7 ± 0.3%
BP + DO	Train	–	–	–	–	98.4 ± 0.4%
	Test	–	–	–	–	87.3 ± 0.5%

*When learning using local errors, the local classifier errors in all layers are reported. Mean and standard deviation from 4 runs. LEL, Local error learning; SYM, Symmetric feedback weights; SCFB, Sign-concordant feedback weights; TLC, Trainable local classifier; DO, Dropout; FA, Feedback alignment; BP, Backpropagation; GI, Gaussian initialization of local classifier weights. For local error learning, local classifier weights were initialized from a uniform distribution, except for cases where GI is indicated. “Fix n” means the parameters of the first n convolutional layers in the network were random and non-trainable*.

In Figure 5, training accuracy can reach 100% as early as the 2nd layer when using a trainable local classifier. However, test error continues to improve until the 6th layer. Perfect training accuracy thus can not be used as a criterion to stop at a particular layer since adding more layers could still improve test-set accuracy. A valid stopping criterion, however, would be to use a validation set to measure the generalization ability of the network and stop using more layers if these layers do not improve accuracy on the validation set. Using a validation set would thus allow us to see that layers above the 6th convolutional layer do not lead to improvements in accuracy, and that we can obtain the most accurate output directly from layer 6.

## 6. Conclusions and discussion

Feedback alignment learning techniques (Baldi et al., 2016; Lillicrap et al., 2016; Nøkland, 2016; Neftci et al., 2017; Samadi et al., 2017) have recently illustrated that weight symmetry between the forward propagation phase and the backward error propagation phase is not strictly necessary for successful learning. This solves one of the core biological plausibility issues of backpropagation which is the need for a weight copying mechanism to realize symmetric feedforward and feedback weights. However, feedback alignment methods, together with multi-layer spike-based learning methods, still make use of non-local errors requiring the network to maintain its state until activity has propagated all the way to the output and a global error signal is generated. Several recent approaches have applied the backpropagation algorithm to learning precise spike times in spiking neural networks (Mostafa, 2017; Zenke and Ganguli, 2017). Yet, errors are still non-local and need to be generated at the top of the layer stack and propagated backwards before any weight updates can take place. The local learning scheme we propose addresses this limitation by generating errors locally in each layer, which gets around the unrealistic requirement of buffering the network activity until a global error is available. Local errors have often been used to augment the top layer errors (Szegedy et al., 2014; Lee et al., 2015). However, until now, relatively little work has been done on supervised learning using exclusively local errors, and none that we know of investigated local error generation using fixed random classifiers.

Our results show that learning using local errors generated using random classifiers, while falling short of the performance of standard backpropagation, significantly outperforms learning using feedback alignment techniques (Baldi et al., 2016; Lillicrap et al., 2016). This holds true even when relaxing the weight symmetry requirement in the local feedback loop and using random fixed feedback weights that are sign-aligned with the random fixed classifier weights in the local learning loop. Maintaining sign-alignment is problematic in the feedback alignment technique as the sign of the feedback weights have to dynamically track the sign of the feedforward weights during training (Liao et al., 2016) which introduces a dynamic dependency between the two sets of weights. In our case, since both sets of weights are fixed, this dependency need only be enforced initially. Dale's law suggests that all outgoing synapses from a biological neuron are either excitatory or inhibitory. This is problematic for current artificial neural networks that use bipolar weights. Dale's law could be reconciled with the bipolar weights typically used in current artificial neural networks by splitting each artificial neuron into two neurons: an excitatory neuron and an inhibitory neuron. Each outgoing connection weight, *w*, coming out of the artificial neuron is also decomposed into the difference of two positive weights, *w*^+^ and *w*^−^ such that *w* = *w*^+^ − *w*^−^. The excitatory neuron projects through weight *w*^+^ to both the excitatory and inhibitory sub-neurons of its target artificial neuron. Similarly the inhibitory neuron projects through the negative weight −*w*^−^. The excitatory and inhibitory neurons in each pair in the network thus always receive the same input and thus always have the same activity which corresponds to the activity of the artificial neuron. The projections of each are either exclusively excitatory or exclusively inhibitory.

Training using local errors attempts to solve many small optimization problems (optimizing the weights of each intermediate layer in order to reduce the error of its local classifier). Thus, in general, it would not converge to the same minima as those reached by standard backpropagation. This is evidenced by the reduced test-set accuracy of local error learning compared to standard backpropagation in our CIFAR10 experiments, which indicates both approaches converge to significantly different optima.

We provide some informal intuition for why we believe learning using local errors gives reasonable performance in practice: the locally generated errors provide a signal to the intermediate layers that encourages them to learn features that are linearly separable across the classification categories. Only by learning linearly separable features can the hidden layer reduce the error of the local linear random classifier. The intermediate layer will typically be unable to learn features that can be used to perfectly disentangle the input into different categories by a linear classifier (as evidenced by the non-zero layer error). However, it is encouraged by the error signal to make the features learned as linearly separable across the training categories as possible. The subsequent layer then builds on these partially disentangled features to attempt to learn even better linearly separable features. Eventually, higher up in the network, a layer is able to learn a disentangled representation that is separable enough to allow the local linear random classifier to achieve adequate performance.

Number of training set errors, even for CIFAR10 converge to zero implying that the local learning algorithm is able to successfully converge to a minimum where training loss is close to zero. Even in the presence of dropout, backpropagation consistently reaches a training error minimum that generalizes better than the minimum reached by local error learning. This is evidenced by the better test set accuracy of networks learned using backpropagation compared to networks learned using local errors, even though both learning mechanisms effectively push number of training errors to zero. We find that while learning using local errors often reaches zero training set error, it lags behind standard backpropagation in test set accuracy. That could be due to the fact that learning using local errors does not simultaneously train multiple level of representations as standard backpropagation. Thus, even though local error learning is training a deep network, it can not truly capitalize on the benefits of depth in improving generalization performance in the same way as backpropagation.

Our CIFAR10, results indicate that locally generated errors allow a convolutional layer to learn good features that are then used by the subsequent layer to learn even more informative features as evidenced by the increased accuracy of the local classifiers in higher layers. In the end, however, our approach solves many small optimization problems where each problem involves only the weights of one layer. We therefore lose one of the core advantages of standard backpropagation learning using a global objective function: the high probability of finding a good minimum in the parameter space that generalizes well when the dimensionality of this parameter space is large, i.e., when it includes all the network parameters (Choromanska et al., 2015; Im et al., 2016). It was thus expected that classification performance will suffer compared to learning using standard backpropagation and a global objective function.

Current state of the art recurrent networks used in various machine learning applications (Hochreiter and Schmidhuber, 1997; Chung et al., 2015) are typically trained using backpropagation through time (BPTT). BPTT suffers from similar issues as backpropagation with regards to biological plausibility and hardware implementation efficiency, namely, the need to buffer activations and neural states across multiple time steps until errors become available. We have described a local learning scheme with spatial locality of error. This scheme could potentially be applied to recurrent networks to generate an error signal at each time step, i.e., the scheme could be used to achieve temporal locality of error. This would be possible if the required recurrent network output at the final time step is known. This final network output could then be used as a virtual target within each time step (not just the final step) to generate a temporally local error. This error does not propagate back through time but is only used to update the weights based on the activity in the current time step. This would greatly reduce the memory requirements of BPTT since activations at intermediate time steps need not be stored, as well as provide both a temporally and spatially local source of error.

Single cell measurements in monkey area IT indicate broad tuning to a range of categories (Sigala and Logothetis, 2002; Kiani et al., 2007). This broad category tuning is realized in the proposed training scheme through the random local classifier weights that define how a neuron contributes to the score of each classification category. During training, the actual tuning properties of each neuron change to be in-line with the pre-defined fixed tuning defined by the random classifier weights, as this is the only way to minimize the local classifier error. Our error generation mechanism has several biologically attractive aspects:

1. It involves only two synaptic projections allowing errors to be generated quickly and weight updates to be carried out before input-induced changes in the states of the neurons have decayed. This avoids the common and unrealistic input buffering requirement encountered in standard backpropagation and feedback alignment techniques.
2. Error generation involves random projections that do not have to be learned. This makes the error generation loop particularly simple and removes any potential problematic interactions between learning the auxiliary classifier weights and learning the main network weights.
3. Strict weight symmetry is not required in the error pathway, only sign-alignment between two sets of fixed random weights is needed.

**Figure 6:**
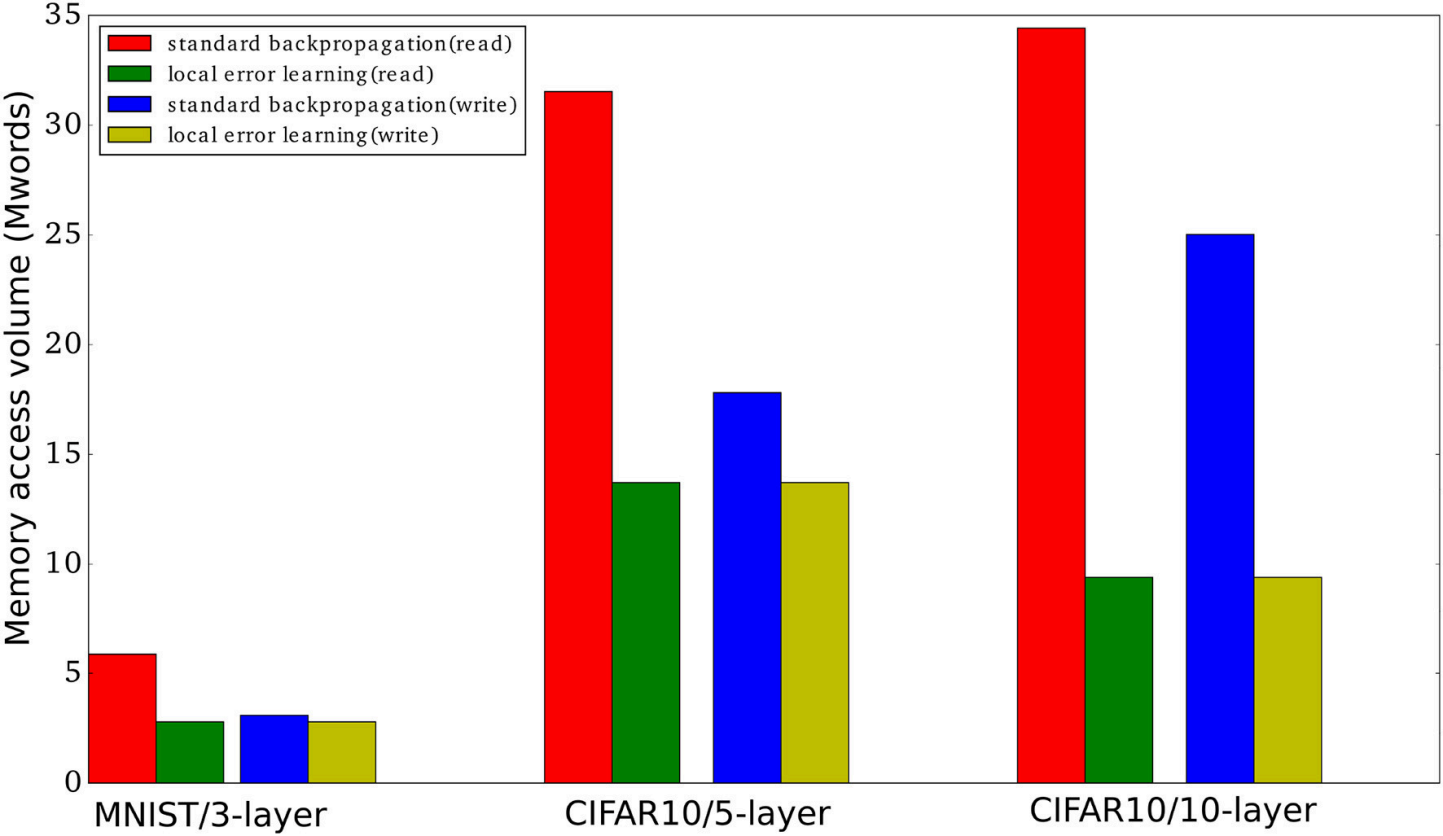
Memory access volume (read and write) when training using local errors and when training using standard backpropagation. We report the memory access volume for the three networks used in this paper when training one mini-batch with 100 examples. The number of parameters, |*P*^*i*^|, for the MNIST network, the 5-layer convnet, and the 10-layer convnet are 2784000, 13716512, and 9402048, respectively. The number of mini-batch activations, |*A*^*i*^|, are 3000 * 100, 40960 * 100, and 156160 * 100, respectively, where 100 is the mini-batch size. We used Table 1 to obtain the read/write volumes.

The use of fixed random local classifier weights allows us to sidestep one of the main hardware-related issues of using auxiliary local classifiers: the need to store the local classifier weights. Especially in large convolutional layers, storing the local classifier weights could be prohibitively expensive in terms of memory resources. Since the local classifier weights need to be accessed in a fixed order during each training iteration in order to calculate the classifier outputs, they can be cheaply, quickly, and reproducibly generated on the fly using a PRNG and a small seed. We have shown that this approach allows us to obtain a learning mechanism that drastically reduces memory traffic compared to standard backpropagation as shown in Table 1. In Figure 6, we show the memory read and write volume needed to train each of the networks presented in this paper for one mini batch with 100 examples. We did not consider the batch normalization parameters since these are typically quite few compared to the number of parameters in the fully connected layers and the convolutional layers.

During inference, the random classifier weights in each layer (which are compactly stored in a small seed) can be used to generate a classification decision during the evaluation of each layer. Thus, if needed, a series of classification decisions can be obtained, one from each layer, at a small computational cost and virtually no memory cost. The decisions from bottom layers, even though less accurate than the decisions from higher layers, can be used in situations where response time is critical. This allows the network to be dynamically truncated where higher layers are not evaluated and the final decision taken from intermediate layers. This feature of the proposed networks enables a dynamical trade-off between accuracy and energy consumption/computational load where only as many layers as allowed by the energy budget, or response time constraint, are evaluated.

## Author contributions

HM developed the main ideas, worked on the simulation experiments, and wrote the paper. VR worked on the simulation experiments. GC wrote the paper.

### Conflict of interest statement

The authors declare that the research was conducted in the absence of any commercial or financial relationships that could be construed as a potential conflict of interest.
